# Prevalence and factors associated with emotional and behavioural difficulties among children living with HIV in Malawi: a cross-sectional study

**DOI:** 10.1186/s12888-019-2046-2

**Published:** 2019-02-08

**Authors:** Fatch W. Kalembo, Garth E. Kendall, Mohammed Ali, Angela F. Chimwaza

**Affiliations:** 1 School of Nursing, Midwifery and Paramedicine, Curtin University, Perth, Australia; 2 Mzuzu University, Mzuzu, Malawi; 3 Kamuzu College of Nursing, University of Malawi, Blantyre, Malawi

**Keywords:** Emotional and behavioural difficulties, Children, HIV/AIDS, Malawi

## Abstract

**Background:**

Approximately 84,000 children under the age of 15 years are living with HIV in Malawi. Although the survival rate of children living with HIV in Malawi has improved due to the increased availability of antiretroviral medications, these children continue to experience numerous challenges negatively impacting on their mental health. The aim of this study was to investigate the prevalence of, and factors associated with, emotional and behavioural difficulties in children aged between 6 and 12 years living with HIV in Malawi.

**Methods:**

A random sample of 429 primary caregivers of children living with HIV drawn from the three main administrative regions of Malawi was recruited in a cross-sectional study. They completed a questionnaire about family socio-demographic characteristics, HIV disclosure, and child demographic and clinical characteristics, as well as the Strengths and Difficulties Questionnaire, Life Stress Scale, Support Function Scale, and Impact on Family Scale which were pre-tested and translated into the local Chichewa language. Data were analysed using descriptive statistics and logistic regression.

**Findings:**

Using the newer band categorisations of the Strengths and Difficulties Questionnaire, parent version, 31% of primary caregivers reported that their child had a slightly raised to very high level of total difficulties. Factors that were associated with difficulties were: primary caregivers’ young age (adjusted odds ratio [aOR] 3.6; 95% confidence interval [CI]: 1.4–9.5); low level of education (aOR 2.6; 95% CI: 1.2–5.7); lack of employment (aOR 2.7; 95% CI: 1.2–5.9); the report of a substantial impact of the child’s illness on the family (3.1; 95% CI: 1.5–6.5); and a low level of family functional support (aOR 2.0; 95% CI: 1.1–4.1). Neither non-disclosure of HIV status nor any of the child demographic or clinical factors were significant in multivariate analysis (*p* > .0.05).

**Conclusion:**

Close to one-third of children living with HIV in this study had high scores indicative of emotional and behavioural difficulties. Emotional and behavioural difficulties in children living with HIV were associated with family demographic and psychosocial factors, but not HIV disclosure. Effective policies and programs that promote the mental wellbeing of children living with HIV in Malawi are indicated.

## I**ntroduction**

UNAIDS reports that there are 1.8 million children under the age of 15 years living with HIV globally; 1.5 million of these live in Sub-Sahara African countries [1]. In Malawi there are approximately 84,000 children under the age of 15 years living with HIV [2]. Although the survival rate of children living with HIV in Malawi has increased due to the increased availability of antiretroviral medications, these children continue to experience numerous challenges that negatively impact on their physical and mental health [3]. These challenges include: high levels of poverty [4, 5]; stigma, discrimination and bullying [6, 7]; the loss of one or both parents [8–10]; food insecurity and poor general physical functioning [11]; and community violence, and harsh physical discipline [12, 13].

The authors of studies conducted in resource-rich countries have identified a high prevalence of emotional and behavioural difficulties, such as anxiety disorder [14–16], attention deficit disorder [16], and depression [14, 17, 18] among children living with HIV. In Sub-Saharan Africa, studies investigating mental health problems in children living with HIV have just started to emerge, and these indicate that the prevalence of mental health problems among children and adolescents ranges between 19% to 52% [7, 19–23]. These studies have identified a number of mental health disorders among children and adolescents living with HIV including: depression and anxiety, [7, 23, 24], social phobia, oppositional defiant disorder, and attention deficit hyperactivity disorder [19].

The terms “mental health” and “mental health problems” are used throughout to convey the meaning that the outcome of interest is health as well as deviations from it. The term “mental health problem” is used synonymously with the terms “emotional and behavioural problem” and “psychopathology”. Psychopathology has been defined as “persistent behaviour, thoughts, and emotions that are likely to impede the accomplishment of developmental tasks necessary for long-term adaptation” ([25] p. 60). Conversely, mental health is defined as “consistent behaviour, thoughts, and emotions that are likely to enhance, or at the least not impede, the accomplishment of developmental tasks” ([25] p. 60).

The burden associated with mental health problems in children living with HIV is significant. The authors of studies conducted in Rwanda and South Africa have reported that children who had depression and conduct problems as well as HIV were signficantly more likely to engage in suicidal behaviours than those who did not have HIV [20, 23]. In a review of studies conducted in resource- rich countries published in 2000, the authors found evidence that adolescents who had HIV and mental health problems were likely to perform poorly at school and to engage in risky sexual behaviours [17]. There is also evidence that children with HIV who have an emotional or behavioural problem are at increased risk of developing a mental health problem during adolescence or adulthood [26]. Furthermore, it has been found that mental health problems accelerate disease progression in children living with HIV by decreasing adherence to antiretroviral medication and lowering body immunity [27, 28]. There is recent evidence that the burden of emotional and behavioural problems is higher in boys compared to girls [30–32]. For example, the authors of a longtudinal study of children living with, or affected by, HIV in South Africa reported that boys had poorer academic performance and more behavioural problems than girls at 12 to15 months follow-up [30].

In 2011, WHO published guidelines for HIV disclosure counselling for children up to 12 years of age [33]. The guidelines recommend that age-appropriate information be given to children as early as possible with full disclosure taking place by the time the child is 12 years of age [33]. Despite this recommendation, many children in Sub-Saharan Africa remain unaware of their HIV status. Primary caregivers have raised concerns over their child’s reaction and the potential impact of disclosure on their mental health [34, 35]. For example, the authors of a qualitative study in Malawi found that caregivers were relactant to tell their child that they had HIV for fear of the negative impact on the child’s mental wellbeing [29]. The few studies that have assessed the relationship between the disclosure of HIV status and mental health outcomes among children living with HIV reveal mixed findings [36–40]. The authors of two cross-sectional studies conducted in United States of America [38, 39], and one prospective cohort study conducted in Thailand [40] have reported that children who knew they had HIV had fewer depressive symptoms compared to those who did not know. In contrast, the authors of a Kenyan cross-sectional study revealed that children who knew their HIV status had more depressive symptoms compared to those who were unaware [36]. Furthermore, the authors of a cross-sectional study conducted in Nigeria reported no difference in mental health problems between children who were aware of their HIV status and those who were not [37].

While children living with HIV in Malawi are at high risk of developing mental health problems, they are unlikely to receive appropriate support from health professionals because of the chronic shortage of material and human resources [41–43]. In Malawi, mental health services are integrated within the general healthcare system with people requiring admission being referred to one of only two tertiary psychiatric hospitals [44–46]. There are few healthcare professionals who specialise in mental health. Therefore, those with mental health problems are most often cared for by health professionals who have limited knowledge and expertise [47, 48]. The authors of a recent study reported that close to 60% of healthcare workers in Malawi who are providing mental health services to people living with HIV are not aware of factors that are associated with the mental health problems these people experience [47]. Moreover, 59% of healthcare workers reported that they did not know how to treat people living with HIV who have a chronic mental health problem [47].

While several studies have examined mental health problems among children and adolescents living with HIV in Malawi and other Sub-Saharan countries, the majority have been conducted with adolescents [7, 19, 21, 23, 49]. Furthermore, despite the WHO recommendation for research to consider the association between non-disclosure of HIV status and mental health among children living with HIV between the ages of 6 and 12 years [50], no previous study has done so. This study was conducted to assess the prevalence of emotional and behavioural difficulties, and associated demographic and psychosocial risk factors, in children living with HIV in Malawi. We hypothesized that emotional and behavioural difficulties in children living with HIV are independently associated with family psychosocial and child demographic and clinical factors. Additionally, we wanted to know if disclosure of HIV status was associated with fewer mental health problems. We hypothesized that children living with HIV who know their HIV status are more likely to have fewer emotional and behavioural difficulties compared to those who do not.

## Methodology

### Study population and design

We conducted a cross-sectional survey involving the primary caregivers of children living with HIV in Malawi. Data were collected in antiretroviral therapy (ART) clinics located in eight randomly selected district hospitals (Mulanje, Nsanje, Mangochi, Salima, Dowa, Kasingu, Mzimba South, and Karonga) in each of the three administrative regions (Southern, Central and Northern). ART clinics were chosen as data collection sites because they were convenient places where the primary caregivers of children living with HIV could be accessed when they brought their children for their monthly or quarterly collection of ART medication. Primary caregivers were eligible to participate if they: were aged 18 years or older, which is the minimum legal age to provide consent to participate in a study in Malawi; had cared for a child living with HIV aged between 6 and 12 years for more than six months; had the ability to provide informed consent; and had not been diagnosed with a psychiatric illness.

Children were not recruited as participants because asking them questions about their health may have resulted in the unintended disclosure of HIV status. Furthermore, the instruments that were used in data collection, including the Strengths and Difficulties Questionnaire (SDQ), were designed to be completed by a parent. While the SDQ is available for children aged around 11 to16 years to self-complete, it was not utilised due to ethical concerns and the relatively small sample of children aged 11 to12 years.

The sample size was calculated on the basis that the rate of HIV status disclosure to children in Malawi may be either high or low. In a recent study conducted in Kenya, Vreeman and colleagues [36] reported disclosure rates among children of 12 and 14 years of age of 44% and 62%, respectively. We used the disclosure rate of HIV, rather than the prevalence of mental health problems, because we did not have a comparison group of children who did not have HIV and we were interested to know if the prevalence of mental health problems varied according to HIV disclosure status. A sample size of 320 was required to provide 90% power to detect a difference in the prevalence of HIV disclosure in children with mental health problems with a 5% chance that a significant difference was due to chance [51]. Assuming a response rate of 75%, approximately 427 potential participants were approached to participate in the survey.

Ethics approval was obtained from the Malawi Government Health Science Research Committee and the Curtin University Human Research Ethics Committee. Informed consent was sought from the study participants prior to data collection. On the clinic day, study participants were recruited using a systematic approach that was trialled during a piloting phase. Potential participants were assigned an odd or even number starting from one, based on the time of their arrival at the clinic. Those assigned odd numbers were screened according to the inclusion/exclusion criteria prior to their participation in the study. A member of the research team who was not part of the clinic staff informed potential participants about the aims, procedures, outcomes, benefits, and risks associated with the study as well as their rights with regards to consent, confidentiality, anonymity, and withdrawal from the study at any point without any compromise or prejudice to the usual care they received at the health facility. Those who consented to participate signed or put their thumbprint on the consent form.

Research assistants with a degree or diploma in Nursing or Public Health were recruited to assist with data collection. Research assistants were trained on the research procedure prior to data collection. The research team was assigned a private room for data collection within each ART clinic. Data collection took place within the waiting time or after participants had been attended to by the healthcare workers. During data collection, children were separated from their primary caregivers to prevent inadvertent HIV status disclosure. Data collection took place from March to July, 2015.

### Data collection instruments

The study questionnaire was translated from English to Chichewa (Malawian local language) and then back-translated to English by language experts following the WHO process of translation and adaption of research instruments [53]. The questionnaire had six sections: (a) family demographic data; (b) child demographic and clinical data (including HIV disclosure status); (c) child emotional and behavioural difficulties; (d) stressful life events (e) impact of illness on the family; and (f) caregiver’s support. All measures were primary caregiver’s report as we only collected data from the primary caregivers of children living with HIV.

#### Primary caregiver demographic and socioeconomic characteristics

In addition to standard demographic data (age, gender, and education) socioeconomic status was measured by the Wealth Index, a tool developed by the World Bank to measure the household socioeconomic status in developing countries on the basis of the household’s ownership of consumer goods, dwelling characteristics, type of drinking water source, toilet facilities, and other characteristics [54].

#### Child demographic and clinical characteristics

The following child demographic and clinical data were collected: age, gender, HIV disclosure status, HIV clinical staging, duration of ART, height, weight, and arm circumference. Body Mass Index percentile was calculated using the Centre for Disease Control Body Mass Index Percentile Calculator for children aged 2 to 19 years old [55]. A BMI for age from the 1st to 4th percentile was categorised as underweight, the 5th to 84th percentile as healthy weight, the 85th to 94th percentile as overweight, and the 95th to 100th percentile as obese [55].

#### Emotional and behavioural difficulties

The SDQ, parent version [56], was used to assess emotional and behavioural difficulties. It is a well-validated instrument, translated in 60 languages including Chichewa (Malawian local language) [57]. The SDQ has 25 items rated on a three-point Likert scale: not true, somewhat true, and certainly true. It has five subscales: emotional problems, conduct problems, hyperactivity, peer relationship problems, and prosocial behaviour. While four of the subscales refer to problem behaviours, the fifth (prosocial behaviour) refers to positive behaviour. Each subscale has five items. The total difficulties score is the sum of scores from all the subscales, except the prosocial subscale, and it ranges from 0 to 40. The scale has adequate internal consistency (Cronbach’s alpha ranging from 0.78 to 0.82) and predictive validity [56].

#### Life stress scale

The Life Stress Scale (LSS), adapted from Tennant and Andrews [58], was used to assess family stressful life events. The adapted scale had the following nine items selected from the broader Life Stress Scale [58]: pregnancy problems, separation or divorce, marital problems, death of a close friend or relative, problems with children, money problems, job loss (involuntary), partner’s job loss (involuntary), and other stressful event. The nine items were selected because, after considerable discussion with researchers in Malawi and Australia it was considered that they were appropriate in the Malawian context. The adapted nine item scale was piloted in Malawi with 10 non-participants and following data collection it was found to have adequate reliability (Cronbach’s alpha 0.71 to 0.76). Primary caregivers were asked to identify all stressful life events that they had experienced in the last year. A binary variable was created by classifying those who experienced less than three stressful life events in one category and those that experienced three or more stressful life events in another category [59].

#### The impact of illness on the family

The impact of the child’s illness on the family was measured by the Impact on Family Scale [60, 61]. It has 24 items, each rated on a four-point likert scale: strongly agree, agree, disagree, and strongly disagree. Total scores range from 15 to 60 with high scores indicating a great impact of the child’s illness on the family [64]. The scale has been found in previous studies to have acceptable internal reliability (Cronbach’s alpha ranging from 0.86 to 0.87) and construct validity [60–63].

#### Caregiver support

The needs of primary caregiver for different types of help and assistance was measured by the Support Functions Scale, short form version [65]. It has 20 items each rated on a 5-point likert scale ranging from (1) “never need this type of support” to (5) “quite often need this type of support”. Total scores range from 0 to 80 with high scores indicating less need for support and low scores indicating a high need for support. The scale has adequate internal consistency (Cronbach’s alpha ranging from 0.77 to .87) and construct validity [65, 66].

### Statistical analysis

Primary caregiver report of their child’s emotional and behavioural difficulties was the primary outcome of the study. The newer band categorisation of the SDQ total difficulties score was used (0–13 ‘close to average’; 14–16 ‘slightly raised’; 17–19 ‘high’; > 19 ‘very high’) [67]. A binary variable was created from the four categories: “close to average” as the reference group and a combination of the last three groups “slightly raised/high/very high” as the problem group [67]. There were three domains of independent variables: family socio-demographic factors, child demographic and clinical factors, and family psychosocial factors. Descriptive statistics were used to summarise the characteristics of the study population. Continuous variables, such as age and duration of taking ART, were recoded into categorical variables. Binary logistic regression was used to estimate independent associations between categorical exposure variables and the binary outcome variable. All exposure variables that were found in bivariate analysis to have a *p*-values of ≤0.25, were entered into the multivariate model [68, 69]. The p-value was set at 0.25 so as not to exclude any variables that may potentially make a significant contribution in multivariate analysis in the presence of other variables [68–70]. There is evidence that selecting variables into multivariate models based on a small p-value cut-off point (usually ≤0.05) in bivariate analysis can result in wrongly rejecting variables that might potentially be important in multivariate analysis [68, 69, 71]. The level of statistical significance was set at *p* < 0.05 in all analyses. Data were analysed using Statistical Software for Social Sciences (SPSS) IBM version 22.

## Results

### Family and child characteristics

A total of 432 primary caregivers from the three administrative regions of Malawi were approached to participate in the study. Three primary caregivers declined to participate and a final sample of 429 primary caregivers was recruited into the study, representing a response rate of 99.3%. The prevalence of family and child characteristics is presented in Table 1. More than half of the primary caregivers (61%) were the biological mothers of children living with HIV, while biological fathers accounted for 15% of the sample. Half of the primary caregivers (50%) were above 40 years of age. Female primary caregivers accounted for 77% of the study sample. More than half of the primary caregivers (78%) had primary school education or higher. With regard to children‘s characteristics, half of the children (50%) were aged between 6 and 8 years, while the remainder were older than 8 years. The proportion of male children living with HIV was slightly higher than female children (52 and 48% respectively). Sixty per cent of children were identified as being underweight. The overall prevalence of non-disclosure of HIV status to children living with HIV was 64%.

**Table 1:** Prevalence of family and child characteristics

Characteristic	N (%)	Characteristic	N (%)
Family Characteristics		Tribe	
District hospitals		Chewa	132 (31)
Mangochi	55 (13)	Yao	74 (17)
Mulanje	53 (12)	Tumbuka	53 (12)
Nsanje	55 (13)	Lomwe	54 (13)
Kasungu	55 (13)	Sena	57 (13)
Dowa	49 (11)	Others	59 (14)
Salima	53 (12)	Occupational status	
Karonga	54 (13)	Work/business	131 (30)
Mzimba	55 (13)	Farming	196 (46)
Region		Looking for a job	29 (7)
Southern	161 (38)	Home duties	73 (17)
Central	159 (37)	Spouse occupational status	
Northern	109 (25)	Work/business	106 (39)
Relationship with the child		Farming	110 (40)
Mother	263 (61)	Looking for a Job	15 (6)
Father	63 (15)	Home Duties	42 (15)
Grandparent	50 (12)	Wealth quintiles	
Others	53 (12)	Poorest	52 (12)
Age of primary caregiver		Poor	43 (10)
18–30	51 (12)	Middle	75 (18)
31–40	164 (38)	Wealthy	78 (18)
41–50	132 (31)	Wealthiest	181 (42)
Above 50	82 (19)	Child characteristics	
Gender of primary caregiver		Age	
Male	99 (23)	6–8	217 (51)
Female	330 (77)	9–10	100 (23)
Marital status of primary caregiver		11–12	112 (26)
Married	273 (64)	Gender	
Single	43 (10)	Male	221 (52)
Widowed	75 (17)	Female	208 (48)
Divorced	38 (9)	WHO HIV clinical staging	
Education level of primary caregiver		Stage I	89 (21)
None	94 (22)	Stage II	80 (19)
Primary	240 (56)	Stage III	219 (51)
Secondary/tertiary	95 (22)	Stage IV	41 (9)
Spouse education level		Nutritional status	
None	47 (17)	Underweight	258 (60)
Primary	134 (49)	Normal	125 (29)
Secondary/tertiary	92 (34)	Overweight/obese	46 (11)
No of children aged ≤12 years		Duration on ARVs^a^	
≤ 2	312 (73)	≤ 1 year	78 (20)
≥ 3	117 (27)	2–3 years	129 (32)
No of children aged 12 years		≥4 years	194 (48)
≤ 2	103 (24)	Disclosure of HIV status	
≥ 3	326 (76)	Yes	156 (36)
		No	273 (64)

^a^Twenty-eight participants are missing in this variable because they were not yet on ARVs despite attending the ART clinic

### Prevalence of family psychosocial characteristics and child emotional and behavioural difficulties

According to the four-band categorisation of the SDQ, higher scores for total difficulties were identified in 31% of the children (see Table 2).

**Table 2:** Child and family psychosocial characteristics

Study measures	Frequency n (%)
Total difficulties score	
Close to average	296 (69)
Slightly raised/high/very high	133 (31)
Emotional problems score	
Close to average	282 (66)
Slightly raised/high/very high	147 (34)
Conduct problems score	
Close to average	269 (62)
Slightly raised/high/very high	160 (38)
Hyperactivity score	
Close to average	354 (82)
Slightly raised/high/very high	75 (18)
Peer problems score	
Close to average	246 (57)
Slightly raised/high/very high	183 (43)
Prosocial score	
Close to average	178 (42)
Slightly lowered/low/very low	251 (58)
Impact of illness on family	
Low level	106 (25)
Significant	252 (59)
Very serious	71 (16)
Level functional support needed	
Low	344 (80)
High	85 (20)
Stressful life events	
< 3	217 (51)
≥ 3	212 (49)

With regard to the subscales of the SDQ, children had higher scores in peer problems, emotional, and conduct subscales, while lower scores were identified in the hyperactivity and prosocial subscales (see Table 2). A significant or serious level of impact of the child’s illness on the family was identified in three-quarters (75%) of study participants. As to functional support, 80% of primary caregivers reported that they needed a low level of support while 20% said that they needed a high level. Almost half (49%) of the participants reported the experience of three or more stressful life events in the family in the previous year.

### Factors associated with emotional and behavioural difficulties in bivariate and multivariate analyses

Table 3 shows the factors associated with emotional and behavioural difficulties in bivariate and multivariate analyses. In multivariate analysis, the odds of a child having an emotional or behavioural difficulty was significantly higher if their primary caregiver was of the Chewa tribe (aOR 2.2; 95% CI: 1.6–12.5) or the Yao tribe (aOR 7.4; 95% CI: 2.5–21.4) compared to those children whose primary caregiver were from the Sena tribe. Primary caregivers who were 30 years of age or younger were more likely to report that their child had an emotional or behavioural difficulty compared to those who were older than 50 years (aOR 3.6; 95% CI: 1.4–9.5). Primary caregivers who were engaged in home duties were 2.7 times more likely to report that their children had an emotional or behavioural difficulty compared to those who were working or conducting a business (aOR 2.7; 95% CI: 1.3–5.9). Primary caregivers who had a primary school education had a higher odds of reporting that their child had emotional and behavioural difficulties compared to those with secondary or tertiary education (aOR 2.6; 95% CI:1.2–5.7).

**Table 3:** Factors associated with emotional and behavioural difficulties in bivariate and multivariate analyses

Variable	Emotional & behavioural difficulties No N (%)	Emotional & behavioural difficulties Yes N (%)	uOR (95% CI)	aOR (95% CI)
Family demographic and psychosocial factors				
Relationship with the child				NI
Grandparent	38 (76)	12 (24)	Reference	
Other relationship	40 (75)	13 (25)	1.0 (0.4–2.5)	
Father	47 (75)	16 (25)	1.1 (0.5–2.6)	
Mother	171 (65)	92 (35)	1.7 (0.8–3.4)	
Age of primary caregiver				
18–30	26 (51)	25 (49)	2.8 (1.3–5.8)*	3.6 (1.4–9.5)*
31–40	117 (71)	47 (29)	1.2 (0.6–2.1)	1.5 (0.7–3.4)
41–50	92 (70)	40 (30)	1.3 (0.7–2.3)	1.5 (0.7–3.3)
> 50	61 (74)	21 (26)	Reference	Reference
Gender of primary caregiver				
Male	78 (79)	21 (21)	Reference	
Female	218 (66)	112 (34)	1.9 (1.1–3.3)*	1.7 (0.8–3.1)
Tribe of primary caregiver				
Sena	50 (88)	7 (12)	Reference	Reference
Other tribes	47 (80)	12 (20)	1.8 (0.7–5.0)	2.1 (0.6–6.9)
Tumbuka	42 (79)	11 (21)	1.9 (0.7–5.3)	2.2 (0.6–7.7)
Lomwe	40 (74)	14 (26)	2.5 (0.9–6.8)	2.7 (0.9–8.3)
Chewa	90 (68)	42 (32)	3.3 (1.4–8.0)**	4.5 (1.6–12.5)***
Yao	27 (37)	47 (63)	12.4 (4.9–31.3)***	7.4 (2.5–21.8)***
Occupation of primary caregiver				
Work/business	104 (79)	27 (21)	Reference	Reference
Farming	128 (65)	68 (35)	2.0 (1.2–3.4)**	1.8 (0.9–3.4)
Looking for a job	22 (76)	7 (24)	1.2 (0.5–3.2)	0.9 (0.3–2.7)
Home duties	42 (58)	31 (42)	2.8 (1.5–5.3)***	2.7 (1.3–5.9)*
Spouse occupation				NI
Work/business	68 (64)	38 (36)	1.3 (0,8-2,2)	
Farming	76 (69)	34 (31)	1.0 (0.6–1.8)	
Looking for a job	9 (60)	6 (40)	1.5 (0.5–4.6)	
Home duties	34 (81)	8 (19)	Reference	
Level of education of primary caregiver				
None	62 (66)	32 (34)	3.6 (1.7–7.5)**	1.9 (0.7–5.1)
Primary	151 (63)	89 (37)	4.1 (2.1–7.9)***	2.6 (1.2–5.7)*
Secondary/tertiary	83 (87)	12 (13)	Reference	Reference
Spouse level of education				
None	24 (51)	23 (49)	2.2 (1.1–4.3)*	
Primary	90 (67)	44 (33)	1.1 (0.7–1.9)	
Secondary/tertiary	73 (79)	19 (21)	Reference	
Number of children < 12 years old at home				NI
≤ 2	220 (70)	92 (30)	Reference	
≥ 3	72 (62)	45 (38)	1.3 (0.8–2.0)	
Number of children > 12 years old at home				NI
≤ 2	66 (64)	37 (36)	1.3 (0.8–2.1)	
≥ 3	230 (71)	96 (29)	Reference	
Marital status of primary caregiver				NI
Widowed	56 (75)	19 (25)	Reference	
Divorced	27 (71)	11 (29)	1.2 (0.5–2.9)	
Married	187 (69)	86 (31)	1.4 (0.8–2.4)	
Single	26 (61)	17 (39)	1.9 (0.9–4.3)	
Wealth quintiles				NI
Poorest	31 (60)	21 (40)	2.2 (1.2–4.3)*	1.2 (0.5–2.9)
Poor	23 (54)	20 (46)	2.8 (1.4–5.7)**	1.9 (0.7–4.7)
Medium	45 (60)	30 (40)	2.2 (1.2–3.9)**	1.7 (0.8–3.6)
Wealthy	58 (74)	20 (26)	1.1 (0.6–2.1)	0.9 (0.4–1.8)
Wealthiest	139 (77)	42 (23)	Reference	Reference
Level of impact of illness on family				
Low level impact	92 (87)	14 (13)	Reference	Reference
Significant impact	174 (69)	78 (31)	2.9 (1.6–5.4)**	3.1 (1.5–6.5)**
Very serious impact	31 (44)	40 (56)	8.4 (4.0–17.4)***	9.4 (1.7–23.8)***
Level of functional support needed				
Low	245 (71)	99 (29)	1.7 (1.1–2.7)*	2.0 (1.1–4.1)*
High	51 (60)	34 (40)	Reference	Reference
Number of stressful life events				
< 3	169 (78)	48 (22)	Reference	Reference
≥ 3	133 (63)	79 (37)	1.8 (1.2–2.7)	1.2 (0.7–2.0)
Child demographic and clinical factors				
Age				NI
6–8	151 (70)	66 (30)	Reference	
9–10	66 (66)	34 (34)	1.2 (0.7–2.0)	
11–12	79 (70)	33 (30)	1.0 (0.6–1.6)	
Gender				NI
Male	147 (67)	74 (33)	1.3 (0.8–1.9)	
Female	149 (72)	59 (28)	Reference	
WHO HIV clinical staging				
Stage I	61 (69)	28 (31)	1.3 (0.6–2.9)	1.8 (0.6–5.2)
Stage II	36 (45)	44 (55)	3.3 (1.5–7.6)**	2.7 (1.0–7.7)
Stage III	169 (77)	50 (23)	0.8 (0.4–1.7)	0.8 (0.3–1.9)
Stage IV	30 (73)	11 (27)	Reference	Reference
Nutritional status				
Underweight	94 (75)	31 (25)	Reference	Reference
Normal	179 (69)	79 (31)	1.3 (0.8–2.1)	1.1 (0.6–2.1)
Overweight/obese	23 (50)	23 (50)	3.0 (1.5–6.1)**	1.5 (0.6–3.8)
Duration on ARVs				NI
≤ 1 year	52 (67)	26 (33)	1.2 (0.7–2.1)	
2–3 years	84 (65)	45 (35)	1.3 (0.8–2.1)	
≥ 4 years	137 (71)	57 (29)	Reference	
Disclosure of HIV status				
Yes	107 (69)	49 (31)	Reference	Reference
No	189 (69)	84 (31)	1.0 (0.7–1.6)	1.1 (0.6–1.9)

***p < 0.001, **p < 0.01, *p < 0.05; aOR adjusted for all variables in the table

In multivariate analysis, the odds of a child having an emotional or behavioural difficulty were higher if the primary caregiver reported a significant level of impact of the illness on the family (aOR 3.1; 95% CI: 1.5–6.5), or a very serious level of impact on the family (aOR 9.4; 95% CI 1.7–23.8) compared to those who reported a low level of impact. Furthermore, primary caregivers who reported a low-level of functional support were more likely to report that their child had an emotional or behavioural difficulty compared to those who reported that they had a high level (aOR 2.0; 95% CI:1.1–4.1).

With regard to child demographic and clinical factors, Table 3 shows that in bivariate analysis, children in WHO clinical stage two (uOR 3.3; 95% CI: 1.5–7.6), and those who were overweight or obese (uOR 3.0; 95% CI: 1.5–6.1) were more likely to have an emotional or behavioural difficulty. HIV disclosure and child demographic and clinical factors were not found to be significantly associated with the children’s emotional and behavioural difficulties.

## Discussion

Nearly one-third of the children living with HIV (31%) were found to have a higher than desirable score for one or more forms of emotional and behavioural difficulty. Primary caregivers reported that 34% of children had an emotional difficulty and 37% had a behavioural difficulty. In addition, it was found that the primary caregiver’s younger age, low-level of education, engagement in home duties, tribe, high level of impact of illness on the family, and low level of functional support were all independently associated with emotional and behavioural difficulties in children living with HIV.

Overall, the prevalence of emotional and behavioural difficulties reported in this study are similar to that reported in Zambian study (29.1%) [52], and lower than that reported in South African study (49.1%) [72]. The difference in prevalence of emotional and behavioural difficulties identified in our study compared to the study conducted in South Africa is likely to be due to the differences in children’s age. While this study targeted the 6 to 12 years old children, the South African study targeted younger children of 3 to 8 years of age [72]. The prevalence of difficulties reported in this study (31%) is higher than that reported in a study conducted among children living with HIV aged between 7 and 13 years in the UK [73].

With regard to the SDQ subscales, more than one-third of participants reported that their children had higher scores (slightly raised, higher, and very high) in the emotional, conduct, and peer relationship problem subscales. The authors of a study conducted in Zambia among children and adolescent living with HIV, reported lower scores in emotional and conduct problems, while the scores in the hyperactivity subscale were similar [52]. The higher scores we found for emotional problems are not surprising, given that this subscale includes complaints of headaches and other physical symptoms, as well as worries, unhappiness, and nervousness which are more likely to be present in children living with HIV. Those who know their diagnosis may wonder about their future health and wellbeing, while those who do not know may wonder what is wrong with them. Children living with HIV are required to take ARV medication every day and some experience side effects. Those who know their HIV status may be resentful and blame their caregivers, while those who do not know may wonder why they have to take the medication when other children do not. The prevalence of conduct problems as measured by the SDQ (38%) was extremely high in this study compared to the 2 % reported in a recent population survey of 4 to11 year old Australian children that also used the SDQ [74]. In the Zambian study, mentioned previously, the prevalence of conduct problems in children living with HIV as measured by the SDQ was 25.2% [52]. While it is difficult to ascertain the exact cause of conduct problems in children living with HIV, there is a great deal of literature that shows that children living with chronic illnesses, such as asthma and Type 1 diabetes, generally have a higher prevalence [75–77]. In addition, 52% of the children were reported to have diminished prosocial behaviour, which is very high in comparison with 10.2% identified in the Zambian study of children living with HIV [52] and 3 % in a UK study of children with HIV [73].

As to the relationship found between the caregiver’s younger age and lower level of education and children’s higher likelihood of having a mental health problem, similar findings have been reported in a study conducted in India, where primary caregivers’ age and educational status were significant predictors of behavioural problems in children living with HIV [78]. In the current study, primary caregivers who had no employment and were doing home duties were also more likely to report that their child had an emotional or behavioural difficulty. Caregivers who are primarily engaged in home duties may spend more time with their children and may be more likely to observe changes in the child’s behaviour compared to those who are employed or doing business. A study conducted in the Netherlands reported that unemployed parents were more likely to report emotional and behavioural problems in their children compared to those who were employed [79]. The authors of a recent Australian study also found that children living with unemployed parents were more likely to have mental health problems than those who were living with employed parents [80]. A lack of parental employment may influence the child’s mental health indirectly by negatively impacting on the psychosocial wellbeing of the parent which in turn affects their relationship with their child [80].

Systematic differences in the level of social support provided to children associated with lineage patterns practiced by different tribes might explain the finding of a higher prevalence of children’s emotional and behavioural difficulties in some tribes compared to others. The Yao and Chewa practice matrilineality while the Sena practice patrilineality [81]. Matrilineal descent is through females where the husband leaves his village and resides at the wife’s village while patrilineal descent is through males and the woman resides at her husband’s village. In both matrilineal and patrilineal societies women assume an inferior position to male members and important healthcare and household decisions are made by men [81]. White (2007) argues that patrilineality provides more social security than the matrilineal system since men feel obliged to take care of their families, in contrast to the matrilineal system where men do not feel obliged to make investments as they feel that they will not live in the village forever [81].

In this study, the prevalence of emotional and behavioural difficulties was not lower for those children who knew their diagnosis. Similarly, the authors of a study conducted in the United States found no difference in emotional and behavioural difficulties between children who knew their HIV status and those who did not [38]. In contrast to these findings, the authors of the Zambian study mentioned previously found that children who were unaware of their HIV status were twice as likely to experience concerning levels of emotional difficulties compared to those who knew their HIV status [52]. However, the participants of that study were children between 11 and 15 years of age who may be more likely than younger children to suspect that something is wrong when they have frequent hospital appointments and are told to take medications [82]. In another cross-sectional study conducted in South Africa among adolescents between 13 and 19 years of age, disclosure of HIV status was a protective factor for depression [23]. As discussed previously, it is not too surprising that children who know their diagnosis experience emotional and behavioural difficulties to the same extent as those who do not. They have reason to feel angry and resentful, as well as to be fearful for their future health and wellbeing. However, there is considerable evidence presented in the WHO disclosure guidelines (2011) that children who are aware of their diagnosis by around 12 years of age adjust much more easily to adolescent and adult roles because they have time to develop adaptive coping strategies [33]. Furthermore, through disclosure children can be informed that their future can be very positive if they continue to take ARV medication and adhere to a healthy lifestyle. The life expectancy for people living with HIV in Australia and other high income countries is now almost on par with the general population [83].

In bivariate analysis we found that children who were less severe in WHO clinical stage two, and those who were overweight or obese were more likely to have an emotional or behavioural difficulty, however, there were no statistically significant relationships in multivariate analyses. No association between severity of HIV infection and mental health problems was found in a study conducted in Tanzania [84]. On the other hand, the authors of cross-sectional studies conducted in Uganda and Malawi have identified stunting and immunosuppression to be associated with poor mental health outcomes [7, 85].

Three-quarters of the primary caregivers reported a significant or very serious level of impact of the child’s condition on the family. This is consistent with the finding from a longitudinal study of children living with HIV in Malawi and South Africa that their mental health was associated with reported burden that HIV placed on the family [12]. This finding also concurs with a recent report that caring for a child living with HIV in Malawi places great financial, physical, and psychosocial hardships on the family [5]. In addition to usual care, the caregivers of children living with HIV must administer medications daily, take the child to hospital clinic appointments regularly, and buy nutritious food that can be costly and in short supply [5, 9]. Over the life course, these children are also more likely to be hospitalised which puts additional strain on the family [9]. Caregivers must also cope with the stress and anxiety associated with knowing that their child has HIV, a potentially life-threatening illness [29]. Coupled with this, caregivers frequently experience stigma and discrimination [5]. Finally, caregivers must manage the complexity of disclosure of HIV status to the child, issues such as the right age to disclose and the potential for disclosure to affect the psychological wellbeing of their child [29]. It is, therefore, not surprising that primary caregivers who reported a significant or very serious level of impact of the illness were respectively three and nine times more likely to report that their child had an emotional or behavioural problem. This finding is in accordance with substantial evidence that childhood mental health problems are generally more prevalent in families that experience psychosocial difficulties [23, 86–88].

Children whose primary caregiver reported that they had a low level of functional support were also more likely to have an emotional or behavioural problem than those where caregivers reported a high level of support. It is a common finding, generally, that the primary caregivers of children who have a chronic illness who have help with human and financial resources cope better with the demands of caring for their child [89]. The results of this study underscore the importance of providing support to the families of children living with HIV.

### Limitations of the study

This study has some limitations. First, multi-informant reports are the preferred way of assessing emotional and behavioural difficulties in children [90]. However, in the current study a single informant report (parent version of SDQ) was used. The parent’s report was based on what they observed in children, and this may have resulted in under or over reporting of the symptoms. Using both teacher-reporting and parent-reporting versions would have helped to validate the findings. As discussed previously, self-report is not recommended for children less than 11 years of age [91]. Furthermore, it would not have been appropriate to ask the children to complete a questionnaire due to the risk of accidental disclosure of HIV status. Although this study relied on parental reporting of the children’s mental health status, it still provides an estimate of the magnitude of behavioural and emotional difficulties among children living with HIV.

Second, this study did not assess the mental health status of caregivers or caregiver’s HIV status. We intentionally excluded primary caregivers who had a history of diagnosed psychiatric problems because we wanted to collect data for the general population only. Furthermore, we did not evaluate mental health status of primary caregivers because it was outside the scope of this investigation, as was collecting information on the HIV status of caregivers. Third, while the sample was drawn from the three administrative regions of Malawi, only primary caregivers receiving care in government hospitals were included. This is unlikely to have resulted in a significant bias because only a very small proportion of families in Malawi can afford private healthcare and the children living with HIV who attend these facilities receive a different level of care. Fourth and finally, as this was a cross-sectional study, we cannot infer causality from the identified relationships.

## Conclusion

In conclusion, this is one of the few population-based studies conducted in Sub-Saharan Africa that has estimated the prevalence of, and identified personal and family risk factors for, emotional and behavioural difficulties experienced by children living with HIV. A considerable proportion of children were found to have one or more emotional or behavioural difficulty. HIV disclosure status was not a risk factor, while family social support was identified as a key protective factor. It is anticipated that the findings will inform mental health policy and the formulation of guidelines for children living with HIV and their families in Malawi and other Sub-Saharan African countries.
